# Leitlinien Österreichische Diabetesgesellschaft: Zusammenfassung der Änderungen*

**DOI:** 10.1007/s00508-023-02194-4

**Published:** 2023-04-20

**Authors:** Michael Resl

**Affiliations:** 1 Abteilung für Innere Medizin, Konventhospital der Barmherzigen Brüder Linz, 4021 Linz, Österreich; 2 Klinisches Forschungsinstitut für Kardiometabole Forschung, Johannes Kepler Universität Linz (JKU Linz), Linz, Österreich

## Diabetes mellitus – Definition, Klassifikation, Diagnose, Screening und Prävention (Update 2023)

Änderungen unserer Leitlinie „Diabetes mellitus – Definition, Klassifikation, Diagnose, Screening und Prävention (Update 2023)“ umfassen die Aktualisierung der weltweit epidemiologischen Kennzahlen für Diabetes, die weiterhin einen deutlichen Anstieg der Anzahl der Menschen mit Diabetes prognostiziert.

Zur diagnostischen Feststellung von T1DM wurde zu den Diabetes-assoziierte Antikörpern ZnT8 hinzugefügt.

Bei Kindern und Jugendlichen kann auch auf Basis eines erhöhten HbA_1c_-Wertes ein Diabetes mellitus diagnostiziert werden.

In Hinsicht auf Diabetesprävention wurden die Risikofaktoren die eine Entwicklung eines Diabetes mellitus begünstigen um Rauchverhalten und Schlafqualität ergänzt. Neue Studienerkenntnisse zur Diabetesprävention von T1DM und T2DM wurden ergänzt.

## Andere spezifische Diabetesformen und exokrine Pankreasinsuffizienz

Neben einer Aktualisierung der medikamentös-induzierten Diabetesformen und jener sekundär im Rahmen anderer endokrinologischer Erkrankungen auftretenden Diabetesformen enthält die überarbeitete Version auch umfassende Information zu Abklärung und Behandlung von monogenetischen Diabetesformen, anderen genetischen Diabetesformen und dem Krankheitsbild der Lipodystrophie. Zudem enthält das Kapitel ein Update zu pankreopriven Diabetesformen und exokriner Pankreasinsuffizienz.

## Antihyperglykämische Therapie bei Diabetes mellitus Typ 2 (Update 2023)

Die Rolle von Metformin als Erstlinientherapie wurde modifiziert, bei Herzinsuffizienz (HFpEF,HFmREF,HFrEF) soll unabhängig vom HbA_1c_ ein SGLT‑2 Hemmer mit positiven Daten verwendet werden. Bei Patient:innen ohne kardiovaskuläre Erkrankung, Herzinsuffizienz oder chronische Niereninsuffizienz wurde das Gewichtsmanagement in den Vordergrund gerückt. Darüber hinaus wurde analog zu den internationalen Guidelines der Beginn einer Injektionstherapie mittels GLP‑1 Analogon noch vor Beginn einer Insulintherapie gereiht.

## Injektionstherapie (GLP1-Rezeptor Agonisten und Insulin) bei Typ 2 Diabetes mellitus

Die Vorteile einer Therapieerweiterung durch GLP1-Rezeptoragonisten werden dargestellt sowie die aktuellen Empfehlungen zur Kombinationstherapieformen.

## Diabetestechnologie (Update 2023)

Die Leitlinien Insulinpumpentherapie und kontinuierliche Glukosemessung wurden neu bewertet und zu einer Diabetestechnologie Leitlinie zusammengefasst. Entsprechend der neuen Evidenz wurden die Empfehlungen für die CGM für alle Diabetestypen modifiziert und dabei wurde auch den neuen Entwicklungen im Bereich der Automated Insulin Delivery Systemen Rechnung getragen.

## Ernährungsempfehlungen für Menschen mit Diabetes (Update 2023)

Im Update 2023 der Ernährungsempfehlungen für Diabetes mellitus wurden nun lebensmittelbezogene Ernährungsempfehlungen angeführt, um bessere praxisbezogene Empfehlungen abgeben zu können. Zudem werden Ernährungsformen wie, low-carb/low-fat, mediterrane Ernährung, Intervallfasten und Formuladiät, in eigenen Kapiteln diskutiert. Abschließend werden Ernährungsempfehlungen bei Diabetes mellitus Typ 1 angeführt.

## Lebensstil: körperliche Aktivität und Training in der Prävention und Therapie des Typ 2 Diabetes mellitus (Update 2023)

Dieser Teil der Leitlinie hebt weiterhin die absolute Notwendigkeit einer lebensstilmodifizierenden Therapie als Grundlage jeder weiteren Therapie in den Vordergrund. Die Fokussierung auf eine Steigerung und Evaluierung der Alltagsaktivität wurde aktualisiert.

## Rauchen, erhitzte Tabakprodukte, Alkohol und Diabetes mellitus (Update 2023)

Während die Prävalenz des Zigarettenrauchens ihren (speziell bei Personen mit Diabetes weit zu hohen) Zenit erreicht hat, ist ein ständig wachsender Trend die Verwendung rauchfreier nikotinhaltiger Produkte, v. a. in Form der elektronischen Zigarette. Aus diesem Grund wurde die frühere Leitlinie „Rauchen und Alkohol und Diabetes mellitus“ mit diesem neuen Thema erweitert. Bzgl. Rauchen und Alkohol erfolgte eine Aktualisierung mit der neuesten Literatur.

## Adipositas und Typ-2-Diabetes (Update 2023)

Die aktuellen Daten hinsichtlich medikamentöser Therapieoptionen im Sinne von GLP-1-Analoga und GIP-GLP‑1 Agonisten wurden aktualisiert.

## Diagnostik und Therapie des Typ 1 Diabetes mellitus (Update 2023)

Neben der Darstellung zur Pathophysiologie und der Insulintherapie bei Typ 1 Diabetes wird auf die Bedeutung der Glukosewerte im Rahmen der Sensormessungen („time in range“) hingewiesen.

## Diabetes mellitus im Kindes- und Jugendalter (Update 2023)

Bei der Therapie des T1D gibt es jetzt automatisierte Insulindosierungs-Systeme (AID), die anhand von kontinuierlichen Sensorglukosewerten mit einem Algorithmus die Insulindosierung einer Pumpe steuern und damit eine bessere metabolische Einstellung erreicht werden. Anhand der Time in Range (Zeit im Zielbereich) kann, besser als mit einem HbA_1c_-Wert, die metabolische Einstellung beurteilt werden. Das Kapitel über den T2D bei Kindern und Jugendlichen wurde um die neueren Therapieoptionen erweitert.

## Gestationsdiabetes Update 2023

Erkenntnisse zur Vitamin D Supplementation zur Prävention von GDM werden erörtert und die Bedeutung der strukturellen antenatalen Interventionsprogramme (Ernährung, Bewegung oder beides) verdeutlicht. Für die kontinuierliche Blutzuckermessung mit FGM und CGM Blutzuckermessystemen wurden Zielbereiche in der Schwangerschaft eingefügt. DASH-, mediterrane und Low Carb-Ernährung zur diabetologischen Betreuung bei GDM werden besprochen und die Vorteile von telemedizinischen Visiten ergänzt. Die medikamentöse GDM-Therapie wurde aktualisiert. Die Bedeutung der Ultraschall-Untersuchungen zur geburtshilflichen Überwachung wird umfassender berichtet und die enterale Intervention bei asymptomatischer Hypoglykämie des Neugeboren überarbeitet.

## Gravidität bei vorbestehendem Diabetes 2023

Schwangerschafts-Outcomes bei Frauen mit präkonzeptionellem Diabetes werden ausführlicher diskutiert. Closed-loop Systeme und die Zielbereiche bei kontinuierlicher Blutzuckermessung wurden ergänzt und die Hypoglykämiewahrnehmung durch Erkenntnisse der CONCEPTT-Studie erweitert. Die Insulintherapie wurde aktualisiert. Bei Retinopathie und Nephropathie wurden Empfehlungen zur Risikoreduktion überarbeitet. Der Einsatz von Aspirin bei erhöhtem Präeklampsie-Risiko wurde aktualisiert, weiters neurologische und makrovaskuläre Komplikationen hinzugefügt sowie die Themen Augenkontrollen, Nierenfunktion, Blutdruck und Lipide aktualisiert.

## Diabetesschulung und -beratung bei Erwachsenen mit Diabetes (Update 2023)

Neben der Wissensvermittlung steht in zunehmendem Maß die Verbesserung von Selbstmanagement im Umgang mit dem Diabetes im Vordergrund der Schulungskurse und Beratungen. Zielvereinbarungen unter Erhaltung der individuellen Lebensqualität bestimmen dazu den Weg. Dies wird durch Beschreibung fünf kritischer Zeitpunkte für den primären bzw. erneuten Einsatz einer Diabetesschulung/beratung unterstützt: Zeitpunkt der Diagnose, Nicht Erreichen der Behandlungsziele im Rahmen der Kontrollen, Umstellung von oraler auf parenterale Therapie, Einsatz neuer Diabetestechnologie, Entwicklung von Komplikationen.

Die Umsetzung adäquater Lebensstilmaßnahmen als Grundlage der Diabetestherapie steht dabei weiterhin im Fokus der Beratung. Spezifisch ethnisch-kulturelle und sozial-strukturelle Aspekte sollten dazu berücksichtigt werden. Zur Behandlung des Typ 2 Diabetes bildet das Disease-Management Programme „Therapie aktiv“ weiterhin ein effektives Mittel zur individuellen, personalisierten Zielerreichung und Prävention diabetischer Folgen und Begleiterkrankungen.

Reviews und Metaanalysen strukturierter Schulungsmodelle geben für die teilnehmenden Personen mit Diabetes signifikante Effekte auf die Senkung des HbA_1c_, des Körpergewichts und des Blutdrucks neben deutlichem Wissenszuwachs an.

Die spezielle Einschulung im Umgang mit neuen Technologien (Smart Pens, Insulinpumpen und Glukosesensor-Systeme, Diabetes Apps) sowie der Einsatz digitaler Medien zur Kommunikation der Betroffenen mit Schulungs/beratungs-Teams gewinnen dabei an Bedeutung.

Viele detailliert ausgearbeitete und spezifisch fokussierte Schulungsprogramme unterstützen dabei Schulungs/beratungs-Teams (z. B. MEDIAS2, HyPOS, SPECTRUM, INPUT) in ihrem Einsatz für die Betroffenen. Selbsthilfevereine, zuletzt verbunden im Dachverein „Wir sind Diabetes“, bieten als Interessensvertretung für Personen in Österreich Unterstützung im Alltag gemeinsam mit Beratung zu spezifischen Themen.

## Blutzuckerselbstkontrolle (Update 2023)

Im Wesentlichen sind die Inhalte dieser Leitlinie unverändert. Inhalte zum Thema CGM finden sich nun jedoch in der Leitlinie „Diabetestechnologie“.

## Individualisierung der antihypertensiven Therapie bei Patient:innen mit Diabetes mellitus. Leitlinie der Österreichischen Diabetes Gesellschaft (Update 2023)

Die Evidenzlage hat sich seit der letzten Version der Leitlinien zur antihypertensiven Therapie nicht grundlegend geändert, neue Empfehlungen verschiedener Fachgesellschaften sind aber publiziert worden. Das individuelle Blutdruckziel ist abhängig von Alter und Komorbiditäten, am wichtigsten bleibt aber für die meisten Patient:innen, dass ein Blutdruck < 140/90 mm Hg erreicht wird. Ergebnisse neuer Studien und Meta-Analysen sowie die aktuellen Empfehlungen der Fachgesellschaften werden diskutiert.

## Lipide (Update 2023)

Das Update der Leitlinie zum Thema Lipide beinhaltet nun auch die therapeutischen Optionen mit Inclisiran und Bempedoinsäure. Ebenso aufgenommen wurde Eicosapentaensäureethylester – wenn auch zur Drucklegung in Österreich nicht erstattbar. Unverändert bleiben die grundsätzlichen LDL-Zielwerte aus dem Online-Update 2020.

## Thrombozytenaggregationshemmung (Update 2023)

Im Wesentlichen sind die Inhalte dieser Leitlinie unverändert. Für Personen mit manifester kardiovaskulärer Erkrankung gilt die Thrombozytenaggregationshemmung als indiziert. Bei hohem oder sehr hohem CV-Risiko sollte sie erwogen werden.

## Diabetische Neuropathie und diabetischer Fuß (Update 2023)

Das ÖDG-Update 2023 der Leitlinie diabetischer Fuß und diabetische Polyneuropathie umfasst zusätzlich die psychologischen Aspekte der Erkrankung und eine detaillierte Empfehlung zur Behandlung der schmerzhaften Polyneuropathie.

Beim diabetischen Fuß (DFS) wurde der Therapie Algorithmus zur Druckentlastung entsprechend den internationalen Empfehlungen spezifiziert, die Klassifikation des DFS angepasst und neue Evidenzen inkludiert.

## Diabetische Nierenerkrankung (Update 2023)

Das gemeinsame Positionspaper der Österreichischen Diabetes Gesellschaft und der Österreichischen Nephrologischen Gesellschaft zur Diabetischen Nierenerkrankung fasst die aktuelle Evidenz zu nephroprotektiven pharmakologischen Wirkstoffen zusammen. Der aktualisierte, multifaktorielle Behandlungsalgorithmus integriert diese rezenten wissenschaftlichen Daten. Es wurde auch Augenmerk daraufgelegt, praxisnahe Empfehlungen auszuarbeiten, in welcher Frequenz Kontrolluntersuchungen stattfinden sollen und wann Überweisungen zu Nephrolog:innen jedenfalls indiziert sind.

## Diagnostik, Therapie und Verlaufskontrolle der diabetischen Augenerkrankung (Update 2023)

Zur Behandlung des diabetischen Makulaödems (DMÖ) sind neue intravitreal zu applizierende Medikamente zugelassen worden. Bei einem der Präparate (Faricimab) handelt es sich erstmalig um einen bispezifischen Antikörper, der nicht nur gegen den vaskulären endothelialen Wachstumsfaktor (VEGF) gerichtet ist, sondern auch Angiopoetin‑2 als Ziel hat, wobei hierdurch eine gefäßstabilisierende Wirkung mit längerer Durabilität des Wirkstoffs erwartet wird. Eine Multizenterstudie hat neue Erkenntnisse gebracht, ab wann Patient:innen mit DMÖ und guter Sehkraft mittels intravitrealen anti-VEGF Präparaten behandelt werden sollen. Weiters wurde in einer Multizenterstudie gezeigt, dass in Patient:innen mit diabetischer Retinopathie ohne Vorhandensein eines DMÖs eine prophylaktische Therapie mittels intravitrealen anti-VEGF Medikamenten zu keinem Visusbenefit längerfristig führt.

## Diabetes mellitus, koronare Herzkrankheit und Herzinsuffizienz (Update 2023)

Die Leitlinie wurde mit den Daten der SGLT‑2 Hemmer in den Patient:innenkollektiven mit HFrEF und HFpEF aktualisiert. Die Notwendigkeit eines regelmäßigen, kardiovaskulären Screenings unterstützt durch Biomarker wie NT-proBNP wurde erneut hervorgehoben.

## Diagnose und Management der Osteoporose bei Diabetes mellitus (Update 2023)

1. Das Frakturrisiko ist bei Diabetes mellitus erhöht und nimmt in Abhängigkeit der Dauer der Erkrankung zu.
2. Die Knochendichte ist in der DXA Messung sehr häufig im osteopenen Bereich, daher darf die DXA Messung niemals die alleinige entscheidungsgrundlage für eine osteologische Therapieentscheidung darstellen.
3. Die Berechnung des Frakturrisikos im FRAX Modell inkl. der Korrekturfaktoren bei Diabetes mellitus werden dargestellt.
4. Osteoporose ist eine chronische Erkrankung mit einer langfristigen Therapieindikation.

## Psychische Erkrankungen und Diabetes mellitus (Update 2023)

Das Positionspapier „Psychische und neurokognitive Erkrankungen und Diabetes mellitus“ Abrahamian et al., wurde im Rahmen des Updates 2023 um zwei rezente Studien ergänzt:

Die Treatment Options for Type 2 Diabetes in Adolescents and Youth Studie (TODAY2 study) zeigt eindrucksvoll die Häufigkeit von psychischen Beeinträchtigungen wie Depression und Essstörungen bei jungen Patient:innen mit in der Jugend manifestiertem Typ 2 Diabetes. Im Verlauf der Erkrankung waren höhere HbA_1c_-Werte, mehr Hypertonie und raschere Progression der Retinopathie mit psychischen Beeinträchtigungen assoziiert (The Today2 Study; Diabetes Care 2022)

In einer schwedischen Register-Studie wurde bei 1.736.281 Teilnehmer:innen gezeigt, dass Menschen mit diagnostizierter Schizophrenie ein deutlich höheres Risiko für Typ 1 Diabetes aufwiesen als Menschen ohne Schizophrenie (HR 2,84). Das Risiko, Typ 2 Diabetes zu entwickeln war für Menschen mit Schizophrenie HR (95 % CI): 13,98 (8,70–22,46), *p* < 0,0001 und für Menschen mit schizoaffektiver Psychose HR (95 % CI): 14.27 (7,36–27,70), *p* < 0,0001 signifikant erhöht (Mizuki Y et al.; Int J Psychopharmacology 2021).

## Therapie der akuten diabetischen Stoffwechselentgleisungen bei Erwachsenen (Update 2023)

### Hyperglykämisch-hyperosmolare und ketoazidotische Stoffwechselentgleisung

Diese Leitlinie gibt klar strukturierte Empfehlungen zur zielgerichteten Akuttherapie lebensbedrohlicher Entgleisungen des Glukosemetabolismus.

## Diabetesmanagement im Krankenhaus (Update 2023)

Die 2023 aktualisierte Leitlinie zum Blutzuckermanagement im Krankenhaus konkretisiert die intravenöse Insulintherapie detaillierter und beschreibt auch den Umgang mit oralen Antidiabetika beziehungsweise GLP1-Rezeptor-Agonisten im Krankenhaussetting näher.

Zudem wurden Empfehlungen zur Verwendung diabetestechnologischer Systeme wie kontinuierliche Glukosemesssysteme und Hybrid Closed Loop Systeme im Krankenhaus in der aktualisierten Version der Leitlinie ergänzt.

## Positionspapier: Operation und Diabetes mellitus (Update 2023)

Im Positionspapier: Operation und Diabetes mellitus (Update 2023) wird der Expert:innenkonsensus zum perioperativen (prä-, intra- und postoperative Phase) Management von Diabetes mellitus, oralen Antidiabetika und Insulintherapie sowie der Umgang mit innovativer Diabetestechnologie beschrieben. Insbesondere das perioperative Pausieren und Reetablieren von oralen antidiabetischen Medikamenten mit potenziellen Nebenwirkungen erfährt Neuerungen: Für das Biguanid Metformin wird bei Menschen mit Diabetes ohne Nierenfunktionseinschränkung ein Pausieren am Operationstag empfohlen, bei Nierenfunktionseinschränkung mit Akkumulationsgefahr weiterhin 24 bis 48 h präoperativ. Bei der Substanzklasse der SGLT2-Inhibitoren soll abhängig von der Operationsdauer, Operationsart und erwartbarer perioperativer Insulindosisreduktion sowie protrahierter Nahrungskarenz ein präoperatives Absetzen von mindestens 48 bis 72 h eingehalten werden, um das Auftreten einer potenziell lebensbedrohlichen euglykämischen diabetischen Ketoazidose zu vermeiden. Im Bereich der Diabetestechnologie wird die Fortführung einer Insulinpumpentherapie und automatisierten Insulinzufuhrsystemen abhängig von Operationsdauer und -art sowie ärztlicher Expertise auf individueller Entscheidungsbasis angeführt und der mögliche, perioperative Stellenwert von kontinuierlichen Glukosemesssystemen beschrieben.

## Therapie der Hyperglykämie bei erwachsenen, kritisch kranken PatientInnen (Update 2023)

Diese Leitlinie gibt Empfehlungen zur Therapie bei kritisch kranken Patienten. Hier ist die Datenlage stabil ohne wesentliche neue Erkenntnisse in den letzten Jahren.

## Geschlechtsspezifische Aspekte bei Prädiabetes und Diabetes mellitus – klinische Empfehlungen (2023)

Neben dem epidemiologischen Update wurde auch die Situation bei Kindern und Jugendlichen näher beleuchtet. Die Bedeutung des OGTTs für die Diagnostik bei Frauen wird hervorgehoben. Beim multifaktoriellen Risikomanagement wird nun genauer auf das kardiovaskuläre Risiko und das schlechtere Risikomanagement bei Frauen eingegangen. Es wird die unterschiedliche Wirkung bestimmter Medikamente zur Behandlung von Fettstoffwechselstörungen genauer beschrieben und auf die besondere Bedeutung der Menopause im Zusammenhang mit dem multifaktoriellen Risikomanagement hingewiesen. Die Primärprävention mit ASS wird umfassender behandelt und um neue Erkenntnisse zu Nettobenefit und Blutungsrisiko ergänzt. Makro- und mikrovaskuläre Komplikationen werden ausführlicher diskutiert, ebenso wie Angststörungen und kognitive Einschränkungen.

## Migration und Diabetes (Update 2023)

Die nachfolgenden Empfehlungen verstehen sich als Ergänzungen zu den generell vorliegenden Leitlinienempfehlungen der Österreichischen Diabetes Gesellschaft und beziehen sich auf Patient:innen mit Migrationshintergrund.

Die allgemeinen Zielwerte und Therapieprinzipen gelten auch bei diesen Personengruppen. Das Erreichen mancher Zielwerte kann aufgrund allgemeiner Barrieren (Sprache, soziokultureller und -ökonomischer Hintergrund, Bildungsgrad etc.) schwieriger sein.

Der Artikel beinhaltet demographische Grundlagen, epidemiologische Besonderheiten, weiters Empfehlungen hinsichtlich der Patient:innenbetreuung und der Diabetesschulung sowie Therapiedosierungsvorschläge während der Fastenzeit Ramadan. Ein weiterer Bestandteil ist die Übersicht über das Ernährungsverhalten und -vorzüge (siehe Ernährungstools) der jeweiligen Kulturen/Regionen, die geographisch unterteilt wurde. Weiteres ist auf die Prävention im Kontext mit der steigenden Prävalenz von Diabetes mellitus bei Menschen mit Migrationshintergrund erwähnt.

Dieser Beitrag wurde in Kooperation mit der Arbeitsgemeinschaft Diabetes und Migration der Deutschen Diabetes Gesellschaft erstellt.

## Geriatrische Aspekte bei Diabetes mellitus (Update 2023)

Beim Kapitel Geriatrische Aspekte bei Diabetes mellitus wird auf die Deeskalation und Vereinfachung der Therapie eingegangen. Individuell festgelegte Therapieziele sollten regelmäßig reevaluiert und angepasst werden, basierend auf chronischer Begleiterkrankungen, der kognitiven Funktion und dem funktionellen Status. Daraus können sich Reduktionen der Dosierung, Umstellungen und ein Absetzen bei anti-diabetischen Medikamenten ergeben. Dies betrifft auch komplexe Insulintherapien. Es sind speziell Vorschläge für eine Vereinfachung der Insulintherapie in Form eines Algorithmus angeführt.

## Diabetes mellitus und Straßenverkehr – ein Positionspapier der Österreichischen Diabetesgesellschaft neu

Das heuer erstmalig in den Leitlinien enthaltene Dokument gibt einen Überblick über Themen der Fahrsicherheit für Menschen mit Diabetes mellitus aus fachärztlicher und verkehrsrelvanter Sicht. Die Prävention und der Umgang mit Hypoglykämien im Straßenverkehr werden diskutiert.

